# Laparoendoscopic single-site surgery for adnexal disease during pregnancy: A single-center preliminary experience

**DOI:** 10.3389/fsurg.2022.994360

**Published:** 2022-10-14

**Authors:** Min Yin, Jiaxin Yang, Huimei Zhou, Xinyue Zhang

**Affiliations:** Department of Obstetrics and Gynecology, National Clinical Research Center for Obstetric and Gynecologic Diseases, Peking Union Medical College Hospital, Chinese Academy of Medical Sciences and Peking Union Medical College, Beijing, China

**Keywords:** laparoendoscopic single-site surgery, adnexal mass, adnexal torsion, pregnancy, ovarian tumor

## Abstract

**Purpose:**

This study aimed to evaluate the safety and efficacy of laparoendoscopic single-site surgery (LESS) in treating adnexal disease during pregnancy.

**Methods:**

Medical records of included patients were retrospectively reviewed and follow-ups of all the patients were performed until the delivery of the fetus. The clinical characteristics, surgical interventions, postoperative complications, and pregnancy outcomes were analyzed.

**Results:**

Six cases were included, with the gestational age ranging from 19 to 31 weeks 1 day. Procedures included salpingo-oophorectomy (*n* = 3), ovarian or paratubal cystectomy with detorsion (*n* = 2), and adnexal detorsion (*n* = 1). The median duration of surgery was 35 min (range, 20–60 min), and the estimated blood loss ranged from 5 to 50 ml. No major intraoperative or postoperative complications were noted. The final pathologic results included high-grade serous ovarian carcinoma, ovarian borderline serous cystadenoma, ovarian simple cyst, endometrioma, and mesosalpinx cyst. Five patients had a spontaneous vaginal delivery at full-term, and one patient had a cesarean section preterm followed by comprehensive staging surgery of ovarian cancer.

**Conclusion:**

Based on the data we included, LESS performed by experienced surgeons appeared to be a safe and feasible alternative to multiport laparoscopic surgery for the management of selected patients with adnexal disease during pregnancy. More studies with large sample sizes at multiple centers are warranted.

## Introduction

Non-obstetrical abdominal surgery is required in approximately 1 in 500 to 1 in 45 pregnant women (1). The most common non-obstetrical abdominal surgeries are for digestive tract diseases such as acute appendicitis and cholecystitis (2). Other conditions with surgical indications during pregnancy include a persistent adnexal mass with potential malignancy or adnexal torsion and abdominal pain, adrenal tumors, splenic disorders, and other rare conditions (3). With the increased adoption of routine prenatal ultrasound, the incidental finding of an adnexal mass in pregnancy has become more prevalent. Most of these lesions are benign and almost invariably resolve by the end of the first trimester. Malignant ovarian masses complicating pregnancy generally involve 1%–8% of cases (4). Surgical intervention is warranted if complications such as ovarian torsion, rupture, or hemorrhage occur; or if there is a strong suspicion of malignancy; or if the mass is large and obstructs the labor tract (5).

Previously, open surgery rather than laparoscopy was preferred during pregnancy due to the concerns for uterine injury and fetal mal-perfusion during laparoscopic surgery (6). With the development of laparoscopic devices and improved surgical techniques, its use is becoming more widely accepted. The Society of American Gastrointestinal and Endoscopic Surgeons (SAGES) and the American College of Obstetricians and Gynecologists (ACOG) both support the use of laparoscopy as a safe, minimally invasive approach for abdominal surgery throughout pregnancy (1, 7). Compared with conventional laparotomy, the advantages of laparoscopic surgery during pregnancy are widely acknowledged: decreased postoperative pain, shorter hospital stay, and early return to normal activity, which is crucial to reduce the risk of thrombotic events during pregnancy (8).

Laparoendoscopic single-site surgery (LESS) is a minimally invasive surgical technique *via* a single incision, which was developed as a less invasive alternative to conventional laparoscopy. Recently, LESS has been widely used in many fields including gynecologic surgery (9). Several advantages of LESS over multiport laparoscopy during pregnancy have been indicated. As the gestational weeks' increase, the uterus becomes larger along with the increased distension of the abdominal wall. In LESS, the approach to the abdominal cavity by an open procedure is much safer, reducing incisional morbidity (10). Furthermore, retrieval of the bulky masses becomes easier through a relatively larger incision. Some studies have suggested that LESS appeared to be a safe and technically feasible treatment for an adnexal mass during pregnancy (11). Although laparoscopy can be performed in all trimesters of pregnancy, data regarding LESS during pregnancy are limited.

Herein, we report a series of six cases who underwent LESS for adnexal disease during pregnancy at our institution. Additionally, we provide a review of studies and cases reported in the literature to evaluate the safety and feasibility of single-port laparoscopic adnexal surgery during pregnancy.

## Materials and methods

### Patients

We performed a monocentric retrospective study of patients who underwent LESS during pregnancy for adnexal disease in the Obstetrics and Gynecology Department of Peking Union Medical College Hospital from July 2018 and November 2021. The gestational age (GA) at the time of surgery was classified into three trimesters: the first trimester from 5 to 14 weeks, the second trimester from 14 to 27^+6^ weeks, and the third trimester from 28 weeks to term. Patients with ectopic pregnancy were excluded. Detailed information associated with the surgical procedure including risks, benefits, and alternatives to multiport laparoscopy *via* the LESS approach was fully discussed with the patients and the informed consent forms of surgery were obtained.

### Surgical procedures

**Figure 1:**
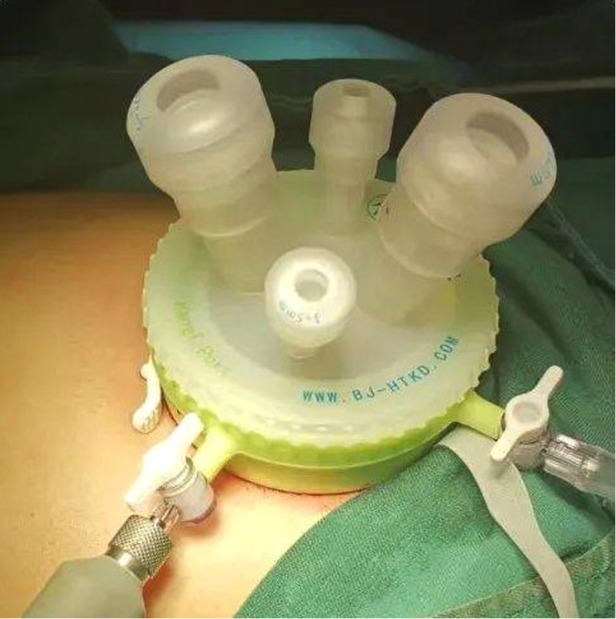
Port for laparoendoscopic single-site surgery.

All surgical procedures were performed by experienced minimally invasive surgeons. Patients were positioned in the supine position with the table tilted to the left to prevent inferior vena cava compression after general anesthesia induction. A Foley catheter was placed and a prophylactic antibiotic was administrated intravenously before incision. The incision was chosen according to the level of the uterine fundus and surgical procedure, in the umbilicus or supra umbilical region. After creating a 2–2.5 cm skin incision, the Hasson technique was performed to avoid injury to the internal abdominal organs and gravid uterus, and a port for laparoendoscopic single-site surgery was placed (Figure 1). A pneumoperitoneum was created with carbon dioxide and the intra-abdominal pressure was maintained at 10–15 mmHg. Finally, the abdominal fascia and subcutaneous tissue were closed using a 1–0 absorbable suture in an “8” closure. The skin was approximated with or without restoration of the umbilical contours and closed with a 4–0 absorbable suture in an intradermic running closure. The fetal heart rate was detected before and after surgery to evaluate the fetal status. Tocolytic drugs were not used prophylactically unless there were signs of threatened preterm labor.

### Data collection and analysis

Medical records of included patients were retrospectively reviewed. Clinical data including age, body mass index (BMI), abdominal surgery history, gravidity and parity history, method of conception, obstetrics complications, GA, and clinical characteristics were collected. The duration of surgery was defined as the interval between skin incision and closure. Intraoperative surgical complications included bladder or bowel injuries and postoperative complications including fever, ileus, and wound infection were carefully documented. The visual analogue scale (VAS) was used to evaluate postoperative pain from 0 to 10. Data relating to surgery including LESS procedure, duration of surgery, estimated blood loss, postoperative 24 h VAS, use of tocolysis, duration of hospital stay, and pathological results were extracted and analyzed. The information on pregnancy outcomes and GA at the time of delivery was confirmed by either medical records in our hospital or by telephone in cases whose prenatal examination or deliveries were at another hospital.

## Case reports

### Three cases of salpingo-oophorectomy

#### Case 1

A 38-year-old woman, gravida 4 para 1, underwent a prenatal examination in her local hospital. At 12 weeks GA, a right adnexal mass was found at routine prenatal ultrasonography. The mass measured 4.8 cm × 3.6 cm and contained papilla with blood flow signals. Surgery was recommended in the early second trimester, but she refused the surgery and chose to close follow-up. Repeat ultrasonography demonstrated that the mass existed consistently and had enlarged to 7.1 cm × 4.2 cm, with an elevated serum CA19-9 129.9 and CA125 230.7 U/ml. Pelvic magnetic resonance imaging also suggested a strong suspicion of malignant ovarian tumor. In order to shorten the operation time and minimize uterine irritability, laparoscopic exploration was performed first to obtain the pathological diagnosis. At 27^+3^ weeks GA, LESS right salpingo-oophorectomy was performed. The right adnexa enlarged to 7 cm × 4 cm and no obvious adhesion was observed. A nodule can be seen on the surface of the right ovary with some yellow fluid. The excised right adnexa including ovarian mass was placed in an Endo bag and exteriorized through the incision. Estimated blood loss was 10 ml, and operative time was 40 min. Frozen-section reported a borderline papillary cystadenoma with focal infiltration. However, the final pathology revealed a high-grade serous ovarian cancer at least stage IC according to the staging classification of international federation of gynecology and obstetrics (FIGO). After a multidisciplinary discussion involving Gynecologic Oncology, Neonatology, Pathology, Anesthesiology, and Maternal Fetal Medicine, adjuvant chemotherapy (carboplatin plus paclitaxel) was given. Then the staging surgery was performed after cesarean section at 33^+3^ weeks GA. At birth, the 2,380 g/44 cm neonate had an Apgar score of 9 points and was transferred to the NICU, but he was discharged without any serious complications. The staging surgery revealed a high-grade serous ovarian cancer at FIGO stage IIIA with the involvement of pelvic lymph nodes. Then the patient received adjuvant chemotherapy and maintenance therapy for ovarian cancer.

#### Case 2

**Figure 2:**
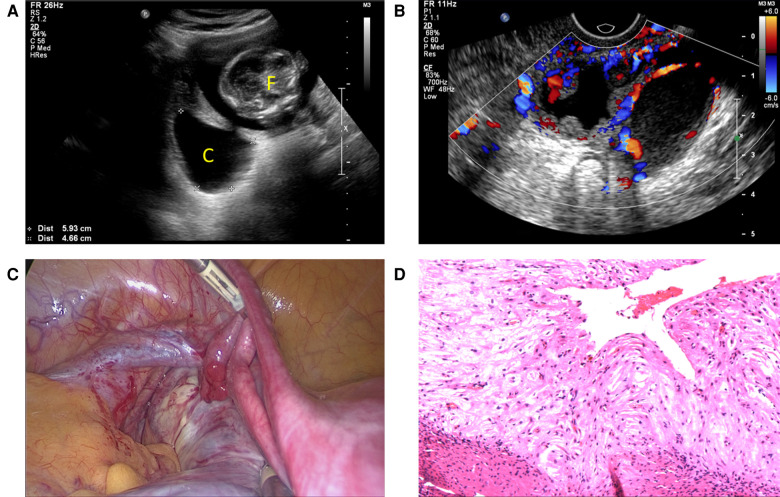
(**A**) Ultrasound graph depicting an intrauterine fetus and an adnexal cyst. (**B**) Doppler ultrasound depicting vascularity within capsule wall and papillary projections. (**C**) Laparoscopy showed an 8 cm left ovarian multilocular cyst adhering to the left pelvic wall and mesorectum. (**D**) Pathologic section showed an endometrioma with extensive stromal decidualization (hematoxylin and eosin, ×200). F, fetus; C, cyst

A 37-year-old pregnant woman, gravida 2 para 1, without any systemic disease, was referred to our hospital at 25^+5^ weeks GA with a persistent left ovarian cyst found by prenatal ultrasound. She got pregnant naturally, and no obstetric abnormality was detected in regular antenatal examinations. A left ovarian cyst, 3.4 cm × 2.5 cm, was noted by an ultrasound examination at 7 weeks GA. Follow-up sonography showed moderate echogenic protrusions in the ovarian cysts, growing gradually. The repeated ultrasonography in our hospital revealed a 4.8 cm × 2.9 cm anechoic area with a clear boundary and another 3.6 cm × 1.5 cm irregular shape with blood flow protruding into the capsule which mimicked a malignant ovarian tumor (Figures 2A,B). The patient decided to undergo LESS left salpingo-oophorectomy at 26^+1^ weeks GA. During the operation, an 8 cm left ovarian multilocular cyst can be seen and the cyst adhered to the left pelvic wall and mesorectum (Figure 2C). We first performed the adhesion lysis and the cyst ruptured with chocolate-like fluid flowing out. Left salpingo-oophorectomy was performed and the specimens were placed in an Endo bag and removed through the single-port incision. Estimated blood loss was 50 ml, and operative time was 60 min, without intraoperative complications. Postoperatively, tocolytics were used due to the presentation of painless uterine contraction. Frozen-section and final pathologic findings confirmed an endometrioma with extensive stromal decidualization (Figure 2D). The patient spontaneously delivered a healthy baby at 38^+2^ weeks GA.

#### Case 3

**Figure 3:**
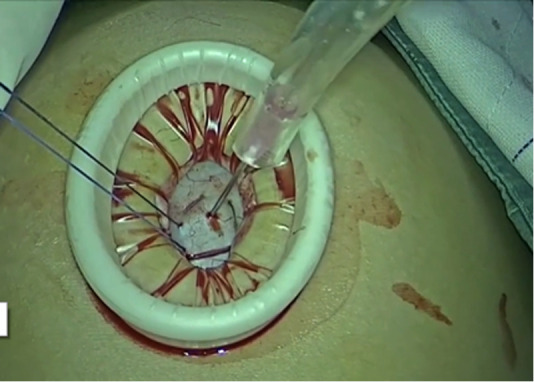
A purse-string stitch was placed around the opening to prevent spillage and the needle was inserted to suck out most of the cystic fluid.

**Figure 4:**
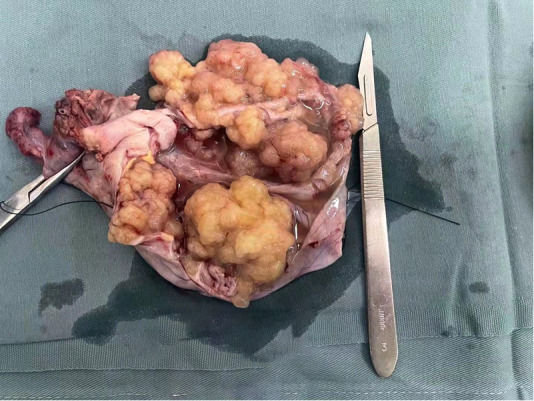
The cyst fluid was clear, and multiple cauliflower-like masses could be seen on the cyst wall.

A 24-year-old woman, gravida 1 para 0, at 8 weeks GA was found to have a big pelvic mass measuring 10 cm in diameter at a prenatal ultrasonographic examination. The mass persisted and grew slowly with serum CA125 67.44 U/ml. At 19^+4^ weeks GA, she was referred to our hospital for evaluation of the big pelvic mass. The repeated ultrasound at our hospital indicated a 9.6 cm × 12.6 cm × 7.8 cm multilocular cystic solid mass and contained papillations and blood flow signal. Given the persistence and increased size of the mass, the patient underwent the surgery in 20 weeks GA. LESS left salpingo-oophorectomy with pelvic washings was performed. The left adnexal mass was grasped using atraumatic grasping forceps and brought to the incision site. A purse-string stitch was placed around the opening to prevent spillage. A needle was inserted to suck out most of the cystic fluid (Figure 3), and the drained adnexal mass was removed through the incision. A thorough inspection of the abdominopelvic cavity revealed no extraovarian disease. Estimated blood loss was 5 ml, and operative time was 40 min. The cyst was dissected and the cyst fluid was clear, with multiple cauliflower-like masses in the cyst wall (Figure 4). Frozen-section and final pathology reported a serous borderline tumor. No tocolytic agent was required and the patient was discharged on postoperative day 2. At 39^+1^ weeks of GA, uneventful vaginal delivery yielded a healthy infant after spontaneous labor.

### Two cases of ovarian/paratubal cystectomy with detorsion

#### Case 4

A 35-year-old woman, gravida 2 para1, was seen for a history of a slowly growing right adnexal mass at 8 weeks GA. She came to the emergency room at 31^+1^ weeks GA because of severe right lower quadrant pain. Pelvic ultrasonography revealed a 5.4 cm × 4.4 cm cyst and a spiral arteriovenous blood flow can be seen. An emergent LESS procedure was performed, and a 5 cm paratubal cyst twisted 360° from the root can be seen. No obvious abnormality is found in the appearance of the right ovary. Therefore, the right paratubal cystectomy was performed. The final pathology demonstrated a mesosalpinx cyst. The estimated blood loss was 5 ml, and the operative time was approximately 30 min. The patient experienced an uncomplicated pregnancy and delivered vaginally at 39^+3^ weeks GA.

#### Case 5

**Figure 5:**
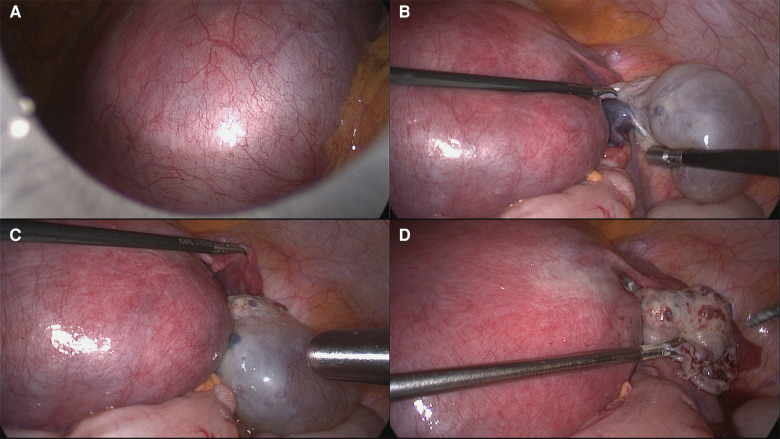
Laparoscopic right fallopian tube detorsion and ovarian cystectomy. (**A**) Enlarged uterus. (**B**) Right ovarian cyst measuring 6 cm × 4 cm without torsion. But the right fallopian tube was rotated 90° counterclockwise and appeared to be dark red. (**C**) The right fallopian tube was reset and the blood flow was restored. (**D**) Reapproximation of ovarian tissue.

A 25-year-old woman, gravida 1 para 0, at 19 weeks GA was seen because of acute right-sided pelvic pain. She conceived naturally without the aid of artificial reproductive technology. A right adnexal mass was discovered at 12 weeks GA *via* prenatal ultrasonography and the persistent mass did not resolve spontaneously. Repeated pelvic ultrasonography revealed a 5.8 cm × 3.9 cm anechoic cyst with a clear and intact margin in the right lower abdomen. No flow was observed at Doppler ultrasonography, and the diagnosis of ovarian torsion was in doubt. An immediate LESS was performed. A right ovarian cyst with a smooth surface was seen. The right fallopian tube was rotated 90 degrees counterclockwise and appeared to be dark red. LESS right fallopian tube detorsion and ovarian cystectomy were performed (Figure 5). Estimated blood loss was 5 ml, and operative time was 30 min. Pathologic analysis revealed a simple ovarian cyst. The patient had no postoperative complications and spontaneously delivered a healthy baby at 38^+5^ weeks GA.

### One case of adnexal detorsion

#### Case 6

A 37-year-old primigravida patient, gravida 2 para 0, was referred at 33^+6^ weeks GA because of severe left lower abdominal pain. Pelvic ultrasonography revealed a 4.6 cm × 2.5 cm cyst with slender partitions in the left adnexal area. No Doppler flow was observed. No extra fluid was noted in the pelvic cavity. The patient complained of lower abdominal pain persistently showing suspicion of adnexal torsion so an emergent LESS procedure was performed. The left ovary showed polycystic enlargement and the left adnexa rotated clockwise 720°. Left adnexa detorsion was performed. There was minimal estimated blood loss, and the operative time was approximately 20 min. The patient experienced no postoperative sequelae and spontaneously delivered a healthy baby at 39^+6^ weeks GA.

## Results

Patient characteristics are summarized in Table 1. Five patients (83.3%) were in the second trimester, and one patient (16.7%) was in the third trimester. The median age of the patients was 36 years (range, 24–38 years), and the median BMI was 23.88 kg/m^2^ (range, 19.33–30.72 kg/m^2^). They were conceived naturally and none of them had a history of abdominal surgery. The median gestational age was 25 weeks (range, 19–31^+1^ weeks). Three patients complained of acute lower abdominal pain and the median mass size measured by ultrasonography was 6.25 cm (range, 4.6–12.6 cm). As for surgical indications, three of them underwent LESS due to adnexal mass with a strong suspicion of malignancy, and the surgical indication for another three patients was suspected adnexal torsion.

**Table 1:** Patients' characteristics.

Case	Age (years)	BMI (kg/m^2^)	Abdominal surgical history	Gravidity and parity history	Method of conception	Obstetrics complications	GA at surgery	Surgical indication	Clinical characterization
1	38	23.74	None	G4P1	Natural pregnancy	None	27^+3^	Suspected ovarian malignancy	Asymptomatic; US: 7.1 cm × 4.2 cm right ovarian cyst contained with septations and papilla. Rich blood flow was detected. Serum CA19-9 129.9 U/ml, CA125 230.7 U/ml.
2	37	25.06	None	G2P1	Natural pregnancy	GDM	26^+1^	Suspected ovarian malignancy	Asymptomatic; US: 4.8 cm × 2.9 cm anechoic area with clear boundary, and another 3.6 cm × 1.5 cm irregular shape protruding into the capsule. Blood flow signals can be seen.
3	24	30.72	None	G1P0	Natural pregnancy	None	20	Suspected ovarian malignancy	Asymptomatic; US: 9.6 cm × 12.6 cm × 7.8 cm multilocular cystic solid mass (papillary) of the left ovary. Serum CA125 67.44 U/ml.
4	35	23.87	None	G2P1	Natural pregnancy	GDM	31^+1^	Suspected AT	Right lower abdominal pain; US: 5.4 cm × 4.4 cm anechoic area in the right lower abdomen. Spiral arteriovenous blood flow can be seen.
5	25	19.33	None	G1P0	Natural pregnancy	None	19	Suspected AT	Right lower abdominal pain; US: 5.8 cm × 3.9 cm anechoic area with a clear and intact margin in the right lower abdomen.
6	37	23.90	None	G2P0	Natural pregnancy	None	23^+6^	Suspected AT	Left lower abdominal pain; US: 4.6 cm × 2.5 cm anechoic area with regular shape and slender partitions. No obvious blood flow was observed.

BMI, body mass index; GA, gestational age; GDM, gestational diabetes mellitus; US, ultrasonography; AT, adnexal torsion.

The surgical procedures and outcomes are summarized in Table 2. The median duration of surgery was 35 min (range, 20–60 min). The estimated blood loss ranged from 5 to 50 ml. The median postoperative hospital stay was 2 days (range, 1–3 days). No major intraoperative or postoperative complications were noted. The median VAS scores for postoperative pain were 3 (range, 2–5) at 24 h after the operation. The final pathologic findings included high-grade serous ovarian carcinoma, ovarian borderline serous cystadenoma, ovarian simple cyst, endometrioma, and mesosalpinx cyst. One patient who underwent adnexal detorsion did not have any specimens. Five patients had a spontaneous vaginal delivery at full-term, and one patient had a cesarean section preterm followed by comprehensive staging surgery of ovarian cancer.

**Table 2:** Patients' surgical procedures and outcomes.

Case	LESS procedure	Duration of surgery (min)	Estimated blood loss (ml)	Postoperative 24 h VAS	Postoperative use of tocolysis	Duration of hospital stay	Pathological diagnosis	Intraoperative and postoperative complications	Pregnancy outcomes
1	Right salpingo-oophorectomy	40	10	4	No	3	High-grade serous ovarian carcinoma	None	CS at 33^+3^ weeks GA followed by ovarian cancer comprehensive staging surgery
2	Left salpingo-oophorectomy	60	50	5	Yes	3	Decidualized ovarian endometrioma	None	VD at 38^+2^ weeks GA
3	Left salpingo-oophorectomy	40	5	2	No	2	Ovarian borderline serous cystadenoma	None	VD at 39^+1^ weeks GA
4	Right salpingo-cystectomy	30	5	3	No	1	Mesosalpinx cyst	None	VD at 39^+3^ weeks GA
5	Right fallopian tube detorsion and ovarian cystectomy	30	5	3	No	2	Ovarian simple cyst	None	VD at 38^+5^ weeks GA
6	Left adnexa detorsion	20	NA	2	No	1	NA	None	VD at 39^+6^ weeks GA

LESS, laparoendoscopic single-site surgery; CS, cesarean section; VAS, visual analogue score; NA, not available; GA, gestational age; VD, vaginal delivery.

## Discussion

With the prevalent use of prenatal ultrasound, the diagnosis of adnexal masses during pregnancy becomes more frequent. Over 90% of functional cysts resolve during gestation and the most common ovarian lesions are benign. Only 2%–5% of persistent adnexal masses are found to be malignant (12). Complications of adnexal masses are usually acute, such as abdominal pain, torsion, and hemorrhagic rupture. Torsion is the most frequent and serious complication, occurring in 25% of cases, typically presenting in the first trimester but can occur during the second and third trimesters as well (13). When symptoms or suspicion of malignancy are present, surgical intervention becomes indicated. Although surgery is often indicated, there are no definitive management guidelines. Nowadays, more and more studies show that laparoscopy is a safe and effective surgical approach in pregnancy, providing several advantages over laparotomy, including reduced postoperative pain, analgesic use, and hospitalization time (14). Cagino et al. (15) investigated the optimal approach to surgical management of adnexal masses in pregnancy based on a meta-analysis of previous studies. They found that laparoscopy was a safe and effective approach for the surgical management of adnexal masses in pregnancy. Laparoscopy was not associated with a statistically increased risk of spontaneous abortion or preterm delivery and may result in a shorter length of hospital stay than laparotomy.

LESS is a one-trocar surgical technique performed through a single incision as a less invasive alternative to conventional laparoscopy. LESS has the advantages of conventional laparoscopy with less postoperative incision pain, shorter hospital stays, and ease of specimen extraction through the umbilical incision (16). Most studies showed that LESS was a safe and feasible technique for gynecologic surgery. However, limited studies evaluate the safety and feasibility of LESS during pregnancy. Here, we present our initial experience with 16 pregnant women who underwent single-port laparoscopic adnexal surgery and assess the safety and feasibility of the procedures. In our study, no major intraoperative or postoperative complications were noted and there was no need for conversion to multiport laparoscopic surgery. Only one patient was delivered preterm for the purpose of comprehensive staging surgery of ovarian cancer. Therefore, LESS appeared to be a safe and technically feasible treatment for adnexal disease during pregnancy.

We searched the published literature on pregnancy-preserving LESS for gynecologic disease on PubMed and 11 studies involving 107 patients were identified. The detailed information of the included literature was summarized in Table 3 (11, 17–26). Traditionally, the recommendation for non-emergent procedures during pregnancy suggests performing surgery during the second trimester in order to avoid surgery during the first and third trimesters to minimize the risk of spontaneous abortion and preterm labor, respectively. Recently, according to SAGES guidelines for the use of laparoscopy during pregnancy, laparoscopy can be safely performed during any trimester of pregnancy when an operation is indicated. In the literature review, the GA at surgery ranged from 4 to 31^+4^ weeks. As for the first trimester, Lee et al. (11) retrospectively reviewed the medical records of 14 women with intrauterine pregnancies who underwent LESS for the treatment of an adnexal mass. Eleven patients were in the first trimester at surgery, and abortion occurred in one case 2 weeks after the operation. In another study reported by Jiang et al. (24), 10 pregnant patients underwent LESS for gynecological acute abdomen in their first trimester. One patient who underwent LESS salpingectomy reported vaginal bleeding at 1 week following surgery and then experienced a spontaneous abortion at 11 weeks GA. In our case series, no one was in their first trimester, and further studies with a larger sample size are needed to evaluate the safety of LESS in the first trimester.

**Table 3:** Published literature studies on LESS for gynecologic disease during pregnancy.

References	Country	Cases	GA at surgery	Surgical procedures	Surgical complications	Obstetric outcomes	Mode of delivery	Neonatal outcomes
Kim et al. (17)	Korea	1	12 weeks	Cystectomy	None	NA	NA	NA
Lee et al. (11)	Korea	14	4 weeks–17 weeks 4 days	Cystectomy (*n* = 9), cyst aspiration (*n* = 2), salpingectomy (*n* = 2), salpingostomy (*n* = 1)	None	FTD (*n* = 10), PTD (*n* = 1), abortion (*n* = 1), lost follow-up (*n* = 2)	VD (*n* = 6), CS (*n* = 5)	NA
Scheib et al. (18)	United States	9	12–21 weeks	Cystectomy (*n* = 6), salpingo-oophorectomy (*n* = 3)	None	FTD (*n* = 7), PTD (*n* = 1), pregnancy ongoing (*n* = 1)	VD (*n* = 7), CS (*n* = 1) secondary to fetal intolerance of labor	NA
Tsai et al. (19)	Taiwan, China	1	14 weeks	Cystectomy	None	FTD	VD	Uneventful
Dursun et al. (20)	Turkey	2	12 weeks; 25 weeks	Cystectomy; salpingo-oophorectomy	None	FTD; PTD	VD; CS (twin pregnancy)	Premature twins were transferred to the NICU
Takeda et al. (21)	Japan	29	10–23 weeks	Cystectomy (*n* = 26), salpingo-oophorectomy (*n* = 2), salpingectomy (*n* = 1)	Threatened premature delivery requiring admission (*n* = 3)	FTD (*n* = 24), PTD (*n* = 4)	VD (*n* = 22), CS (*n* = 6), termination of pregnancy with clear cell carcinoma followed by debulking surgery (*n* = 1)	Uneventful
Xiao et al. (22)	United States	13	10 weeks 2 days–28 weeks 1 day	Cystectomy (*n* = 6), salpingo-oophorectomy (*n* = 2), myomectomy (*n* = 1) salpingectomy (*n* = 1); cerclage placements (*n* = 2), paratubal cystectomy (*n* = 1).	None	FTD (*n* = 5), PTD (*n* = 4), lost follow-up (*n* = 3), pregnancy ongoing (*n* = 1)	VD (*n* = 4), CS (*n* = 5)	Uneventful
Takeda et al. (23)	Japan	1	31 weeks 4 days	Salpingectomy	Threatened premature delivery requiring admission	FTD	VD	Uneventful
Jiang et al. (24)	China	26	First trimester (*n* = 25), Second trimester (*n* = 1)	Salpingectomy (*n* = 11), cystectomy (*n* = 15)	None	FTD (*n* = 21), PTD (*n* = 4), abortion (*n* = 1)	VD (*n* = 21), CS (*n* = 5)	Uneventful
Guan et al. (25)	United States	1	16 weeks 3 days	Cystectomy	None	Pregnancy ongoing	NA	NA
Han et al. (26)	China	10	8 weeks 2 days–18 weeks 2 days	Cystectomy (*n* = 10)	None	FTD (*n* = 9), PTD (*n* = 1)	VD (*n* = 4), CS (*n* = 6)	NA

LESS, laparoendoscopic single-site surgery; GA, gestational age; NA, not available; FTD, full-term delivery; PTD, preterm delivery; VD, vaginal delivery; CS, cesarean section.

Limitations of LESS in previous studies included restricted instrumentation because of the clashing of devices and limited surgical vision. Nowadays, several technical improvements have been suggested. Single-site laparoscopic ovarian cystectomy can be very challenging in pregnancy, especially when the need for suturing arises. Since the utero-ovarian ligament tissues are usually overstretched during pregnancy as the uterus enlarges, selected adnexal masses could be exteriorized. Exteriorizing the ovary and cyst after intraperitoneal drainage may allow for extracorporeal suturing that is faster and easier. Kim and Kwon (17) first reported LESS for exteriorization and cystectomy of an ovarian tumor in week 12 of the pregnancy. Concerns about spillage of cystic contents were raised, especially contents of suspected malignant masses. To prevent tumor rupture and spillage during the cystectomy, Guan et al. suggested a combination of in-bag and extracorporeal ovarian cystectomy as a novel alternative minimally invasive approach (25).

As for postoperative management, the routine use of prophylactic tocolytics is under discussion. In a study evaluating the safety and feasibility of gasless LESS in the management of adnexal masses during pregnancy (21), three intramuscular doses (5 mg/body, 8 h apart) of isoxsuprine hydrochloride were routinely administered as prophylactic tocolysis for 3 days in the postoperative period. Xiao et al. (22) claimed that tocolytics are not required postoperatively for prophylaxis in those who are not experiencing uterine contractions based on their experience. According to SAGES guidelines, there is no evidence to support the routine use of prophylactic tocolytics. However, these drugs may be indicated when signs of preterm labor are present. In our case series, tocolytic drugs were not routinely used prophylactically and only one patient used tocolytics postoperatively because of signs of uterine contraction.

The impact of laparoscopy during pregnancy on obstetric outcomes is conflicting. Some studies reported non-favorable pregnancy outcomes, including a moderate increase in the risk for preterm delivery and lower birth weight, while others argue for a non-inferior pregnancy outcome (27, 28). According to the case–control study from Solomon et al., there was no difference between the groups regarding adverse pregnancy outcomes, including rates of preterm birth. However, a higher rate of cesarean sections among the laparoscopy group was noted and most of the indications for cesarean section were elective, possibly due to the physician's and patient's lower threshold for cesarean section (29). When it comes to LESS during pregnancy, Han et al. reported 10 cases who underwent single-port laparoscopic adnexal mass removal during pregnancy: only one patient delivered at 34^+4^ gestational weeks, while other patients delivered after a full-term pregnancy. As for the delivery mode, four patients delivered naturally, and six had a cesarean section delivery (26). Takeda et al. compared the maternal and neonatal outcomes in the LESS surgery group and the multiport laparoscopic surgery group. No significant differences in obstetrical complications, mode of delivery, and neonatal outcomes between the two groups were shown and the neonatal course was uneventful in all infants. Preterm delivery was noted in four cases in the LESS surgery group including two cases of monochorionic-diamniotic twins, a case of pregnancy-induced hypertension, and a case after treatment for threatened premature delivery (21). Another study also concluded that there was no apparent association between preterm delivery and abortion with single-port laparoscopic adnexal surgery. Pregnancy outcomes of the cases included in their study were generally acceptable and 10 of 14 cases were delivered at term (11). In our case series, only one patient had cesarean section preterm followed by comprehensive staging surgery of ovarian cancer, while the rest of the patients had spontaneous vaginal delivery at full-term.

Here, we presented our initial experience of single-port laparoscopic adnexal surgery and assessed the safety and feasibility of the procedures. However, our study also has weaknesses, namely, its retrospective nature and limited sample size. Additionally, bias caused by a single-center analysis may also have influenced the findings of the study.

## Conclusion

In conclusion, based on the data we included, LESS performed by experienced surgeons appeared to be a safe and feasible alternative to multiport laparoscopic surgery for the management of selected patients with adnexal disease during pregnancy. More studies with large sample sizes at multiple centers are warranted for further assessment of the utility of LESS during pregnancy.

## Data Availability

The original contributions presented in the study are included in the article/Supplementary Material, further inquiries can be directed to the corresponding author.
